# On the Development of Implicit and Control Processes in Relation to Substance Use in Adolescence

**DOI:** 10.1007/s40429-015-0053-z

**Published:** 2015-04-08

**Authors:** Reinout W. Wiers, Sarai R. Boelema, Kiki Nikolaou, Thomas E. Gladwin

**Affiliations:** 1 Addiction Development and Psychopathology (ADAPT) Lab, Department of Psychology, University of Amsterdam, Weesperplein 4, 1018 XA Amsterdam, The Netherlands; 2 Department of Cultural Diversity and Youth, Utrecht University, Utrecht, The Netherlands; 3 Research Centre-Military Mental Health, Ministry of Defense, Utrecht, The Netherlands

**Keywords:** Adolescence, Substance use, Brain development, Dual-process models, Review, Attentional bias, Approach bias, Memory bias, Executive functions, Cognitive control, fMRI, Alcohol, Marijuana, Cigarette use

## Abstract

Adolescence is a period in which brain structures involved in motivation and cognitive control continue to develop and also a period in which many youth begin substance use. Dual-process models propose that, among substance users, implicit or automatically activated neurocognitive processes gain in relative influence on substance use behavior, while the influence of cognitive control or reflective processes weakens. There is evidence that a variety of implicit cognitive processes, such as attentional bias, biased action tendencies (approach bias), memory bias and at a neural level, cue reactivity, are associated with adolescent substance use. The impact of these implicit processes on the further development of addictive behaviors appears to depend on moderating factors, such as (premorbid) executive control functions. Clear negative effects of adolescent substance use on executive control functions generally have not been found using behavioral tasks, although some studies have identified subtle and specific effects on cognitive functioning.

## Introduction

Dual-process models emphasize the importance of both impulsive and reflective processes in many behaviors in the development of addiction [1•, 2, 3•]. From this perspective, with continued substance use, implicit or automatically activated processes (e.g., cue reactivity, attentional bias, approach tendencies, and memories in response to drug-related stimuli) gain in relative control over substance use behavior, while the moderating influence of reflective processes (e.g., thinking about long-term negative outcomes) on the addictive behavior weakens. Reflective processes require both the ability to moderate impulses (executive control functions) and motivation to do so, which is related, for example, to alternative goals in life that are incompatible with continued heavy substance use [1•, 2, 3•]. A central function of executive functions is to shield long-term goals from temptations with short-term benefits but long-term negative outcomes [4••].

Normative adolescent brain development may contribute to a propensity to engage in risky behaviors, such as substance use [5]. In general, motivational processes develop relatively quickly during adolescence, while cognitive control processes develop in a more gradual way [5]. Several studies have shown that brain maturational changes in the prefrontal cortex continue well into late adolescence [6–8]. This normative developmental discrepancy has tentatively been related to increased risk taking and substance use in adolescence (e.g., [5]). For example, with the onset of puberty, erotic stimuli suddenly become motivationally relevant [9•]. Some youth (e.g., with a family history of substance use) may show deficits in cognitive control prior to substance use, which put them at higher risk for substance use (e.g., [10]). Importantly, a recent review emphasized the importance of social and affective brain processes in adolescence [11••], which relates this normative increase in risk taking and substance use in adolescence to the importance of social goals rather than to a lack of ability to moderate impulses to use. In addition, social controls such as parental monitoring and supervision help to constrain adolescent risk-taking behavior [5].

A number of reviews have proposed neurocognitive models in which this temporary maturational discrepancy between impulsive motivational processes and cognitive control processes is prolonged or exaggerated by heavy substance use during adolescence [3•, 12, 13, 14••]. In short, these models predict that as a consequence of heavy substance use during adolescence, cue reactivity and related cognitive biases in attention, memory, and action tendencies would become stronger and exert a stronger effect on subsequent substance use, while the development of executive control functions would be relatively delayed (or ultimately impaired), resulting in more cue-driven behavior. These effects may be enhanced as a result of acute effects of the substance on both priming motivational processes and impairing control processes (see [15] for a review). Here, we review the effects of substance use on the hypothesized strengthening of cue-induced motivational responses or biases in implicit cognitive processes. We then discuss effects of substance use on executive control processes. Finally, we discuss the interplay between implicit cognitive processes and executive control processes in relation to substance use behavior.

Before we begin the review, we discuss two caveats. First, it should be noted that dual-process models have been criticized for different reasons, including the neural implausibility of separate motivational and control neural systems [16], and because such models can easily incorporate motivational homunculi (i.e., how did the control system learn about the benefits of long-term goals?). We see current dual-process models as heuristic models at a higher level of description, representing emergent properties of underlying neural processes that interact and unfold over time [14••], cf. [17•]; these underlying processes must be modeled to banish the motivational homunculus out of typical dual-process models [18••]. Second, the models outlined generally focus on only two types of neurocognitive processes in relation to addiction: exaggerated motivational processes (cue reactivity and related processes) and impaired cognitive control processes. However, other neurocognitive processes have also been implicated in the development of addiction, such as interoceptive processes involving the insula [19], negative reinforcement, and allostasis [20••], and of course, social processes are very important in adolescent addictive behaviors too [21]. This review not only focuses on human studies involving the most commonly used substances during adolescence: alcohol, cannabis, and cigarette smoking but also includes reference to problem gaming behavior [22] and reactivity to food cues [23].

## Substance-Related Cognitive Biases and Adolescent Substance Use

A number of studies have investigated the relationship between implicit cognitive processes and adolescent substance use (Table 1). Implicit cognitive processes are thought to develop rapidly after substance use initiation; it is unclear to what extent they also play a role in the initiation of substance use (as explicit expectancies do, see [2, 3•]). Most studies were cross-sectional and focused on one cognitive bias and its relation to adolescent addictive behaviors. However, some studies were prospective, which permitted examination of predictive effects of cognitive biases over time, in relation to the development of the addictive behavior. We distinguish between three commonly assessed cognitive biases [2, 3•]: attentional bias, biased action tendencies (approach bias), and memory biases. We also considered measures of general impulsivity or of general cognitive control when they were included, but we excluded studies that did not use measures of substance-related cognitive biases (e.g., studies measuring impulsivity and cognitive control, but not cognitive biases, e.g., [24]). While most studies focused on one cognitive bias, some larger prospective studies examined the effects of multiple cognitive biases on substance use, hence these studies will figure in multiple sections (e.g., [25]).

**Table 1:** Cognitive Bias and Adolescent Substance Use

Study reference	Subst	Subjects	Design (cross/prosp)	AtB	Mem assoc	ApB	General measures	Main findings
Field et al. [33]	Alc	91 (16–18-year olds (M = 16.8)), 92 % male	Cross	Emo stroop			Delay discounting (DD) heavy > light	DD and AtB correlated^a^; DD and AtB also correlated with drinking variables.^a^
Pieters et al. [26]	Alc	195^a^ (12–16-year olds (M =13.7)), 44 % male	Cross	VPT				AtB only related to alcohol use in OPRM1 g-carrying adolescents
Cousijn et al. [27]	Can	53 (18–30-year olds (M = 24-25)), 34 % male	Cross	Emo Stroop			Classical Stroop	AtB in heavy cannabis users, not in controls; no difference in classical Stroop. In heavy users, AtB associated with Can use, not moderated by EC.
Van Hemel-Ruiter et al. [28•]	Alc	86 (12–18-year olds (M = 14.9)), 43 % male	Cross	VPT			Reward and punishment sensitivity (SPSRQ) and EC: flanker	Reward sensitivity related to alcohol use, not to AtB. AtB related to drinking in weak EC, not in strong EC participants
Van Hemel-Ruiter et al. [29]	Alc, Can, Smok	682 (14–18-year olds (M = 16.1)), 49 % male	Cross	SOT AtB				Engagement toward reward (500 ms) associated with alc and can use; engagement toward non-punishment (250 ms) cues related to smoking.
Zetteler et al. [39]	Alc	30 (15–20-year olds (M = 18), 57 % male), 15 COAs	Cross	Emo Stroop (sub- and supraliminal)			Classical Stroop	AtB for alcohol for supraliminal Stroop, correlated with anxiety and low alcohol use. No effect/difference on classical Stroop.
Van Holst et al. [22]	Game	92 (12–17-year olds (M =15.1)), 100 % male	Cross	VPT and Emo Stroop			Go/NoGo	No relationship AtB (VPT) with level of problem gaming; but relation with errors in Emo Stroop (not RT).
Pieters et al. [25]	Alc	427 (12–16-year olds (M = 14.0)), 48 % male	Prosp (1 year)	VPT	IAT; word assoc	SRC	WM: SOPT	Alcohol use at T2 not predicted by implicit cognition measures or WM. positive-arousal IAT interacted with positive expectancies to predict change in alc use.^b^ SRC predicted alc use when negative expectancies were low.^c^
Larsen et al. [30]	Smok	125 (13–18-year olds (M = 16.1), 79 NL and US); 67 smoke	Cross	Emo Stroop	bIAT	AAT	Classical Stroop; WM: SOPT BART	Stronger differences for nationality than between smoke/non-smoke (ApB). Smoking correlated positively with risk-taking (BART) and negatively with WM. Nicotine dependence correlated with AB and weak inhibition.
Janssen et al. [31]	Alc	378 (12–18-year olds (M = 14.9)), 35 % male	Prosp (6, 12, 18 months)	Emo Stroop VPT	bIAT	AAT SRC	SURPS; delay discount (DD)	T1 VPT predicted alc consumed T2, T3, T4; T1 SRC predicted alc consumed T2 and drink status T2 after controlling for T1; Emo Stroop predicted alc T3. DD and SS (SURPS) predict drinking T4. Biases not predicted by use.
Willem et al. [32]	Alc	94 (15–21-year olds, (M = 18.0)), 52 % male	Cross	VPT		SRC	Self-report attentional and inhib control	ApB predicted AUDIT scores in boys only. Low AtB and high attentional control were related to low AUDIT scores.
Peeters et al. [41]	Alc	374 (M = 13.6), 88 % male, spec. educ	Cross			AAT	Classical Stroop	Alcohol ApB related to alcohol use, especially youth with poor inhibition.
Peeters et al. [42•]	Alc	374 (M = 13.6), 88 % male, spec. educ	Prosp (6 months)			AAT	Classical Stroop	Alcohol ApB related to change in alcohol use (6 months), in youth with poor inhibition.
Pieters et al. [43]	Alc	238 (12–16-year olds (M = 13.8)), (50 % male)	Cross			SRC	WM: SOPT and alcohol-specific parenting	Gender × parenting (rules) × ApB predicted alc use: in boys with permissive parents, ApB predicted alcohol use (no WMC moderation).
Van Hemel-Ruiter et al. [44]	Alc	43 (13–17-year olds, (M = 15.1)), 51 % male	Cross			SRC (irrel. feature) and AAT	WM: random number generation	Positive valence of alcohol in picture rating task and alcohol avoidance responses (AAT) were related to alcohol use.
Cousijn et al. [45•]	Can	73 (18–25-year olds (M = 21-22)), 66 % male	Prosp (6 months)			AAT		Heavy cannabis smokers showed cannabis ApB, but controls did not. ApB was predictive of escalation of cannabis use 6 months later.
Ames et al. [52•]	Alc Can	467 at-risk 14–19-year olds (M = 16.7), 57 % male	Cross		Word assoc; Pict Assoc; phrase compl		Personal (imp, SS) dissociat exp	Mem Assoc was a strong predictor of Can and Alc use. Use and dissociative experiences predicted substance use problems. Sensation seeking predicted Mem Assoc for Alc and Can.
Ames et al. [53]	Can	121 high-risk adol, 15–19-year olds (M = 16.7), 64 % male	Cross		Word assoc; IAT; EAST		WM SOPT; SS; explicit exp	WAT, IAT-excitement, relaxation and negative expectancies predict unique variance in Can use.
Belles et al. [54]	Alc	128 (12–24-year olds (M = 15.2)), 20 % male	Cross		Excite IAT		COA (CAST) explicit exp	COAs showed stronger alcohol-excitement associations than controls. Both IAT and expectancies related to drinking.
O’Connor et al. [55]	Alc Smok	378 (10–12-year olds (M = 11.1)), 48 % male	Cross		SC-IATs valence × subst			Stronger negative alcohol associations in those who never tried alcohol.
Grenard et al. [56•]	Alc Smok	145 high-risk, (M = 16.7), 66 % male	Cross		Word assoc		WM: SOPT	Word assoc x WM predicted alc and cigarette use: weaker WM related to stronger relationship of automatic associations with substance use
Sherman et al. [61•]	Smok	10-18-year olds, (M = 13.4), 51 % male and parents	Prosp (18 months)		IAT		Parents’ IAT and explicit attitudes	Implicit, not explicit attitudes, predicted smoking initiation. Youths’ implicit attitudes were predicted by mother’s implicit and explicit attitudes
Thush and Wiers [58]	Alc	*N* = 100, *n* = 51 12-year olds (57 % male); 49 15 years (41 % male)	Prosp (1 year)		4 SC-IATs: pos, neg, arousal, sedation		Explicit exp (same 4 scales)	Explicit negative and implicit positive and arousal associations predicted binge drinking at follow-up. Negative explicit expectancies negatively predicted binge drinking in 15-year-old boys. Positive associations predicted binge drinking in 12-year-old boys.
Thush et al. [57]	Alc	88 (14–20-year olds (M = 16.3)), 58 % male	Prosp (1 month)		EAST; IAT; word assoc		Explicit exp; SS; personality	Explicit negative expectancies and word associations predicted later alcohol use.
Thush et al. [59]	Alc	88 (14–20-year olds (M = 16.3)), 58 % male	Prosp (1 month)		EAST; IAT; word assoc		WM (SOPT)	In low WM group, positive-arousal associations predicted alc use; in high WM group explicit exp predicted alc use.
Van der Vorst et al. [60]	Alc	608 (11–17-year olds (M =13.9)), 43 % boys	Prosp		Word assoc		Parental alc	Parental alcohol use (T1) predicted adolescent alcohol use (T2), with partial mediation by aldolescents’ memory associations (T1).

*Subst* substance, *AtB* attention bias, *ApB* approach bias, *M* mean, *EC* executive control, *Alc* alcohol, *Can* cannabis, *Smok* smoking, *COA* children of alcoholic parent(s), c*ross* cross-sectional, *Prosp* prospective, *Emo Stroop* emotional (substance) Stroop test, *EAST* extrinsic affective Simon task, *AAT* approach avoidance task, *IAT* implicit association test, *bIAT* brief IAT, *Sc-IAT* single-category IAT, *VPT* visual probe test, *SRC* stimulus response compatibility task, *SOT* spatial orienting task, *DD* delay discounting, *RT* reaction time, *BART* balloon analog risk task, *SURPS* substance use risk profile scale, *AUDIT* alcohol use disorders identification test, *EAST* extrinsic affective Simon task, *CAST* children of alcoholics screening test, *word assoc* word association test, *Pict Assoc* picture association test, *Mem assoc* memory association, *phrase compl* phrase completion test, *pos* positive, *neg* negative, *imp* impulsivity, *SS* sensation seeking, *dissociat exp* dissociative experiences, *explicit exp* explicit expectancies, *WM* working memory, *SOPT* self-ordered pointing task, *irrel feat* irrelevant feature, *SPSRQ* sensitivity to punishment and sensitivity to reward questionnaire

^a^Study 1, subset of Pieters et al. [25] who provided genetic data

^b^Combination of high positive expectancies and strong positive-arousal associations (IAT) predicted escalation

^c^Combination of low negative expectancies and strong alcohol-approach tendencies (SRC) predicted escalation

### Attentional Biases and Adolescent Substance Use

We identified 11 studies on attentional biases in adolescence [22, 25–27, 28•, 29–33, 39] sometimes stretching into young adulthood (Table 1). One study [27] only included young adults (age 18+) and was the only study on attentional bias for cannabis. Nine studies were cross-sectional, two were prospective, and most (8 of 11 studies) focused on alcohol.

Most studies assessed an attentional bias with a substance Stroop task or a Visual Probe Task (VPT). In a substance Stroop task [34], participants color-name substance-related and neutral words and an attentional bias is defined as an interference effect (slower reaction time (RT) and/or more errors with substance words). In the VPT [35•], two stimuli are presented at the same time (e.g., alcohol picture on one side of the screen, water on the other side), after which a probe (e.g., an arrow pointing up or down) appears on one of the two sides that the participant needs to react to (typically with a button press). Attentional bias on the VPT is calculated as a facilitation effect: faster RT when the probe replaces the substance compared with the control-picture.

Among older adolescent drinkers, an attentional bias (Stroop) was positively related to level of alcohol consumption [33]. However, in most studies this association was qualified, and the association was only found in subgroups: e.g., in OPRM1 g-allele carriers [26], or in adolescents with relatively weak executive control only [28•]. In addition, a combination of a relatively weak attentional bias (VPT) for alcohol and strong attentional control was associated with low levels of drinking in adolescence and young adulthood [32]. We note, however, that the VPT has been found to have a rather poor reliability [36]. In two prospective studies, one study found the VPT to be predictive of the development of drinking in subsequent waves [31], while the other study did not [25], which may reflect VPT reliability issues. Recently, more reliable scoring algorithms for VPT have been proposed, which focus on variance rather than mean differences [37•, 38] and may help to reduce inconsistent results in future VPT research.

Alcohol can disproportionally bias attention for various reasons, such as its appetitive motivational properties, but also for its threatening properties, which are particularly salient in specific populations, such as children of alcoholics ([39], cf. [40]). In a large representative sample of youth, Van Hemel-Ruiter and colleagues [29] used a general spatial orienting task, in which participants reacted to cues which can signal likely reward (e.g., win points for a possible prize) or punishment (e.g., need to repeat the task until a minimum score is reached). Scores for attentional engagement and disengagement were derived, both for cues of reward and punishment at short (250 ms) and longer delays (500 ms). Attentional engagement toward reward at long delay was found to be uniquely and positively associated with substance use (alcohol and cannabis), while attentional engagement to non-punishment (short delay) was positively associated with smoking. These findings confirm the notion that an attentional bias in adolescent substance users is generally of an appetitive nature (expected reward or non-punishment), while in special populations such as children of alcoholic parents, this may be different.

Regarding other addictive behaviors, young adult cannabis users demonstrated an attentional bias (Stroop) not observed in non-users, and within cannabis users the attentional bias was found to be positively related to cannabis use [27]. In adolescent cigarette smokers, attentional bias (Stroop) was positively related to nicotine dependence scores [30]. In adolescent problem gamers, the pattern of association with attention bias and gaming behavior only emerged in the errors and not in reaction times in a game Stroop [22]. Although research on attentional bias and addictive behaviors other than alcohol is limited, emerging studies suggest a pattern of findings similar to alcohol, in which attentional bias is positively associated with addictive behaviors.

In sum, studies of attention bias in adolescents provide tentative evidence that adolescent substance users and problem gamers show an attentional bias for their substance or gaming behavior (with most of the evidence for alcohol use). However, there is also evidence that this association may be most relevant in a high-risk subgroup, defined either by relatively weak control or strong appetitive responses. Findings for other addictive behaviors, such as cannabis and cigarette use, are preliminary. This field of research is plagued by measures with relatively poor reliability, although this problem is targeted by recent research.

### Approach Biases and Adolescent Substance Use

We identified nine studies on approach biases in adolescence [25, 30–32, 41, 42•, 43, 44, 45•], sometimes stretching into young adulthood. Again, one cannabis study with young adults only [45•] was included. Eight studies assessed an approach bias for alcohol or smoking, five were cross-sectional, and three were prospective (see Table 1).

All studies examining approach biases in relation to adolescent substance use either used a variety of the substance—approach avoidance task (AAT) [46•], or a variety of the stimulus response compatibility (SRC) task [47•]. There are important differences between these tasks. In the AAT, participants react by moving a joystick toward or away from themselves, and a zoom effect makes the picture increase or decrease in size, to create a sense of approach or avoidance, respectively. Usually (but not necessarily, see [48•]), the AAT is used with irrelevant feature instructions [49•]. Irrelevant feature instructions direct the participant to react to a feature of the stimulus other than its content, typically the format of the picture (e.g., instructions to “pull” pictures in landscape-format and “push” pictures in portrait-format). The approach bias is then calculated in the AAT as the difference in RT for pulling vs. pushing pictures of the substance, sometimes relative to baseline differences in pushing and pulling for neutral objects [45•]. In contrast, the SRC uses a symbolic approach or avoidance movement (a matchstick figure or manikin approaches or avoids the substance-stimulus or neutral stimulus on the screen) and is typically used in a relevant feature paradigm (see [44] for an exception). In a relevant feature paradigm, the instructions are more explicit: in one block, participants are asked to move the manikin toward the substance (and away from neutral pictures), and in another block, participants move the manikin away from the substance (and toward neutral). Approach bias is then calculated in the SRC as the difference score between these blocks. An advantage of relevant feature tasks is that their reliability is typically higher [50]. However, these tasks are less implicit in the sense of indirect measurement—potentially outside awareness of participants, since the instructions do not refer to the contents of the pictures [49•, 51].

Peeters and colleagues [41] administered the alcohol-AAT, and a classical Stroop test as a moderator (representing cognitive control), in 374 young high-risk adolescents from special education (primarily boys with externalizing problems). Among drinkers, an alcohol-approach bias was positively related to concurrent alcohol use [41], and this was moderated by cognitive control (stronger relationship between alcohol-approach tendencies and drinking in low control individuals). In the same sample, escalation of drinking 6 months later was predicted by the baseline approach tendencies in low cognitive control individuals only [43]. In a normative sample of youth (*N* = 238), it was found that an approach bias for alcohol (SRC) was related to heavy drinking in boys with permissive parents only [43]. In a partly overlapping prospective sample [25], approach bias (SRC) predicted escalation of alcohol use only in adolescents with weak explicit negative expectancies. Another large prospective study found that approach bias measured by the SRC, but not with the AAT, predicted prospective alcohol use through 18-month follow-up [31]. Finally, regarding alcohol, one cross-sectional study found an approach bias for alcohol to be related to alcohol use and problems in adolescent and young adult men, but not women [32]. Another small study (43 adolescents) found a stronger alcohol avoidance response to be related to heavier alcohol use but used an atypical measure [44]. Taken together, these studies generally indicate a positive relationship between an alcohol-approach bias and drinking or changes in drinking, although the association appears to be stronger in certain subgroups (e.g., boys, or subgroups of boys without strong control, either internally or externally—rules by parents).

Regarding other substances, one cross-national study, which compared smoking and non-smoking Dutch and American adolescents, found stronger differences in approach bias (AAT) between countries than between smokers vs. non-smokers [30], highlighting that cultural context matters with indirect measures. Finally, an approach bias for cannabis was found in young adult heavy cannabis smokers (AAT), but not in controls, and the approach bias predicted escalation of cannabis use over 6-month follow-up [45•]. The limited extant research suggests a positive association between approach bias and cannabis, but not for cigarette use.

The pattern of results for approach bias is similar to that for attentional bias: most studies find a positive relationship between an alcohol-approach bias and drinking (or changes in drinking). However, there are issues in the measurement of approach bias and also indications that the association between approach bias and substance use may only hold for specific subgroups (e.g., boys with permissive parents, boys in special education, adolescents with low explicit negative expectancies). Findings regarding other substances are still preliminary with positive findings for cannabis and negative for smoking.

### Memory Associations and Adolescent Substance Use

We identified 13 studies [25, 30, 31, 52•, 53–55, 56•, 57–60, 61•] from 11 datasets on memory biases in relation to substance use in adolescence (see Table 1). Two studies [57, 59] report on the same dataset, as do two other studies [53, 56•]. Seven studies were prospective. We excluded studies on children that did not predict subsequent substance use [62, 63] and intervention studies (e.g., [64, 65]).

Most memory association studies used either a version of the RT-based measure of associations, a variety of the implicit association test (IAT) [66•] or a variety of an open-ended memory association task. Open-ended memory tasks generally ask for the first response to an ambiguous word (e.g., “draft”) or picture, sentence completion (e.g., first association to “Friday night, feeling good…”), or picture associations. An overview and theoretical background for these memory association measures can be found in Stacy et al. [2]. Two studies directly compared these memory association measures in adolescents, one in American high-risk adolescents in relation to cannabis smoking [53] and one in Dutch adolescents in relation to alcohol use [57]. Both studies found open-ended memory associations to be a better predictor of substance use than other implicit measures of alcohol-related cognitions.

Memory association tasks assessing implicit alcohol cognitions have been found to prospectively predict alcohol involvement in youth, over and above explicit alcohol expectancies. For example, implicit arousal associations and explicit negative alcohol expectancies (e.g., alcohol will make me feel sick) predicted onset of binge drinking in 12-year olds and escalation of binge drinking in 15-year olds [58]. There was some indication that explicit negative alcohol expectancies were especially important in 15-year-old boys, and that implicit arousal associations were salient in 12-year-old boys [58]. In a large prospective study, Van der Vorst and colleagues [60] found that implicit memory associations predicted alcohol use one year later, with partial mediation of the effect of parental drinking by the child’s association. One study of adolescents and young adults found relatively strong alcohol-excitement associations in children of alcohol-dependent parents [54].

Memory associations as indicators of implicit cognitions also appear to predict cannabis and tobacco use. In a large cross-sectional study [52•], memory associations were predictive of both alcohol and cannabis use. With regard to smoking behavior, Sherman and colleagues [61•] found that young adolescents’ implicit (IAT), and not explicit, attitudes predicted onset of smoking, and these associations were in turn predicted by the mother’s implicit and explicit attitudes toward smoking. Another study, which parsed the IAT in underlying components using mathematical quad-modeling [67•], found that 10–12-year-old children who did not try to smoke had stronger negative associations than those who did try to smoke [62].

Two studies tested the hypothesis from dual-process models that implicit associations would predict addictive behaviors more strongly in adolescents with relatively weak working memory, one using open-ended memory associations [56•] and another study the IAT [59]. Both studies found confirmatory evidence: stronger prediction of memory associations of concurrent smoking and drinking [56•] and of short-term prospective alcohol use [59]. Note that the same finding of an interaction between implicit cognition and working memory was reported for the impact of action tendencies (approach bias) on concurrent and prospective drinking in high-risk adolescents [41, 42•]. However, this interaction was not confirmed in two prospective samples with normative samples [25, 31]. For example, Pieters and colleagues [25] reported that positive-arousal associations (IAT) predicted prospective alcohol use in interaction with explicit positive alcohol expectancies, such that an escalation of alcohol use in the subsequent year was only observed in adolescents with both relatively positive implicit and explicit expectancies.

In sum, a number of studies have found that implicit memory associations predict concurrent and prospective use of different substances (alcohol, smoking). There also are some indications of parental influences on these associations: parental substance use (alcohol [60]) and parental implicit and explicit attitudes (smoking [61•]). In this regard, positive-arousal associations could be related to familial risk for alcoholism [54], as has been found for explicit alcohol expectancies [40]. Regarding other sources of individual differences in substance associations, adolescents’ personality (sensation seeking), has been found to be associated with implicit memory associations in high-risk adolescents [53], although this was not confirmed in a recent study with a normative sample [31]. Similarly, two studies with high-risk adolescents found that implicit memory associations were particularly predictive of substance use in adolescents with relatively weak cognitive control capacity (working memory [56•, 59]), as has been found for high-risk adolescents’ action tendencies (approach bias) [41, 42•], but this was not confirmed in normative samples [25, 31]. Thus, the existing literature supports an association between implicit memory associations and adolescent substance involvement (concurrent and prospective), but there are mixed findings regarding moderation of the association by executive control.

### Neurocognitive Studies of Cue Reactivity in Adolescent Substance Users

There are as yet few studies on the neural mechanisms of cognitive biases in adolescents, let alone studies directly investigating effects of age on such mechanisms. However, a number of cue-reactivity studies have examined the neural response to cues associated with addictive substances (alcohol, cigarette, cannabis) and food in adolescents. We therefore first briefly review what abnormalities in adolescents’ cue reactivity may tentatively suggest about neural mechanisms thought to be involved in cognitive biases.

Overall, fMRI studies in adolescents and young adults show that addictive substance cues evoke, as expected, increased activation in brain regions associated with salience and the reinforcement of behavior. In particular, medial and orbitofrontal prefrontal cortex, insula, anterior cingulate gyrus, amygdala, and the striatum appear to be over-stimulated by alcohol-related [68•, 69], smoking [70], and food [23] stimuli. This network of regions is involved with the encoding of value, salience, and the use of value information in response selection and adjustment of behavior and cognitive states [71]. Further, cue reactivity within this network is correlated with various real-life risky behavior, such as desire to drink and drinks per month [68•], drinking escalation [69], cigarettes smoked per day and self-reported smoking addiction [70], and food craving [23]. Thus, the increased activation of this adaptation-to-reinforcement network suggests that drug cues indeed automatically evoke abnormal attentional and response selection processes.

However, to the best of our knowledge, studies using implicit measures-style tasks to probe automatic drug-related neural processes are almost absent in adolescents, and also still very scarce in adults. Some very recent studies have adapted existing tasks to include an implicit component [72, 73•] or used existing implicit measures in the scanner [74–76]. Research in our lab has also started to adapt implicit measures specifically for use in EEG [72] and fMRI [77•] designs. Some of these tasks have been used to study neural responses to alcohol cues in various task contexts and their relationship to adolescent drinking behavior and escalation of alcohol use.

In the first paper from this line of research in adolescents [78•], adolescents (age 16–20) who reported at least one full drink in their lifetime performed an alcohol-Go/NoGo task during EEG measurement, in which effects of alcohol cues and acute effects of alcohol consumption were studied. Alcohol cues caused an increased need for inhibition as indexed by the N2 component, but acute alcohol consumption reduced the magnitude of this component, possibly reflecting the loss of protective inhibition. Further, the acute effect of alcohol on the error-related negativity (ERN), an ERP component reflecting adaptive processes that occur in response to making an error, during blocks of alcohol cues, was shown to be related to escalation of drinking over six months. Subjects whose ERN was less blunted by alcohol consumption were more at risk of escalation. This appears to reflect a sensitivity effect similar to low level of response [79], in which subjects who are strongly affected by negative alcohol effects are less likely to abuse it.

As yet, to our knowledge, this is the only directly relevant study on neural activity measured in an implicit measures task in adolescents. The following studies on alcohol and marijuana do involve neural effects in implicit measures tasks, but in young adults aged around 18 to mid-20s.

We know of four quite recent neurocognitive studies involving alcohol. First, in an EEG study, effects of alcohol cues and the acute effects of alcohol consumption (high or low alcohol dose vs. placebo) on neural responses in an adapted alcohol-AAT were tested [72]. Beta-band desynchronization of the posterior EEG was used as a measure of preparatory neural activity. Preparatory processes were found to be enhanced for approach-alcohol actions, and this effect was enhanced by acute alcohol consumption. These results suggest that alcohol cues, or more generally addictive stimuli, evoke an action tendency in the sense of Frijda’s [80] states of action readiness. Second, neural activation related to attentional bias was explored with a novel cued visual attention task [77•]. Alcohol distractors were found to evoke activation in the precuneus, and subjects with weaker activation in the precuneus were more likely to show risky drinking behavior. Further, subjects were found to be faster to shift their attention away from alcohol than from control distractors. These results were tentatively interpreted to suggest a protective role of attentional shifting when confronted with potentially distracting alcohol stimuli. Third, brain activation related to (in)congruence with automatic associations has been studied using an alcohol-valence IAT [76]. Heavy drinkers showed increased positive associations with alcohol, and only in heavy drinkers compatible vs. incompatible trials showed increased activation in, especially, the dorsal striatum and the insula. Fourth, in an alcohol-Go/NoGo task performed during fMRI measurement, heavy vs. light drinkers showed increased activation of the insula on beer-cued NoGo trials [73•]. Thus, these studies show that there is a neurocognitive “reactivity” not just to cues by themselves, but to those cognitive contexts that evoke the alcohol-related automatic processes posited by dual-process models.

Marijuana use in young adults is associated with changes in activation during a marijuana-relaxation IAT [74]. Notably, dorsal striatum activation, related to habit formation [81], was higher in the congruent vs. incongruent condition in marijuana users, similarly to the above finding in an alcohol IAT. In another study, neural effects of a marijuana approach bias (i.e., activation differences found for an approach-marijuana/avoid neutral vs. avoid-marijuana/approach neutral contrast) were measured using a stimulus response compatibility (SRC) task [75]. Although there were no group differences between heavy and light users, within the heavy users, approach bias-related activation in a widespread set of regions, including amygdala, insula, inferior and ventromedial prefrontal cortex (PFC), and precuneus, was found to co-vary with lifetime cannabis use. Also within the heavy users, escalation of problem severity at a 6-month follow-up measurement was found to be predicted by reduced activation of dorsolateral prefrontal cortex and anterior cingulate cortex during congruent vs. incongruent blocks, which the authors interpreted in terms of a protective role of regulatory self-control and performance monitoring.

In summary, neurocognitive studies involving implicit measures have only just started to map out how the brain reacts to conflicts between cognitive control and automatic tendencies, and how such effects are related to the development of addictive behaviors in adolescence. Results so far do support roles for mechanisms of habit, attention shifting, error processing, and approach tendencies, in line with dual-process models. However, the role of executive control is as yet under-studied; it would be informative for it to be explicitly included as a moderator in future studies. Another important future direction for research is to relate the cue reactivity of the network for adaptive, reinforcement-based behavior to abnormal bias-related activation (as in [82]) as this could further empirically connect the theories of incentive salience and dual-process models (cf. [83]).

## Effects of Adolescent Substance Use on Executive Functions

The following sections review behavioral research, followed by neurocognitive research, on effects of adolescent substance use on neuropsychological measures and neurocognition. The review focuses on alcohol, given limited research on effects of other substance use on executive functioning in adolescents.

### Behavioral Studies on Effects of Alcohol on Executive Functions

In a recent review [84•], neurocognitive performance in adolescent alcohol users is described. The authors conclude that alcohol-consuming adolescents display poorer performance on tasks measuring a wide variety of cognitive domains, where the amount of impairments appears to be positively related to the number of drinking days. Furthermore, the authors suggest that post-drinking symptoms (i.e., hangover and withdrawal) might be more harmful for the adolescent drinking than the quantity of alcohol intake, since higher levels of such symptoms have found to be associated with poorer learning and memory performance. Another focus of research has been more complex cognitive functioning, such as risky decision-making, where young drinkers are found to be more sensitive to rewards.

A drawback of above-mentioned review is that no distinction is made between heavy drinking and alcohol use disorder (AUD). This is problematic since, although AUD is a serious and impairing disorder, the majority of alcohol drinking youth do not meet the criteria for the disorder. Furthermore, it is unclear if, and to what extent the presence of a psychiatric disorder (e.g., conduct disorder) has a confounding effect on the relationship between alcohol use and maturation of cognitive functioning. In another review [85•], this distinction is made more clearly, indicating that there have been relatively little studies specifically focusing on binge drinking, but available studies indicate less optimal performance on a diffuse set of cognitive domains.

Taken together, the above-mentioned studies indicate a diffuse pattern of worse cognitive functioning in heavy drinking adolescents and adolescents with AUD compared with healthy controls, with little consistency across studies. A major drawback of these studies is that they are cross-sectional in nature, hampering the possibility of drawing causal inferences. To the best of our knowledge, only three studies have investigated the effects of alcohol use on maturation of neurocognitive functioning in a longitudinal design, two large studies (described in: [86]) and one small study [87].

In a recent large prospective study (*n* = 2230) [86], six drinking pattern-groups were identified: non-drinkers, light drinkers, infrequent heavy drinkers, increasers, decreasers, and chronic heavy drinkers. Chronic heavy drinking adolescents had been drinking five to six glasses or more on a single occasion every weekend during the past 4 years. These groups were compared on a set of computerized RT tasks, assessing working memory, inhibition, and sustained and shift attention. In contrast with what was expected, there were no significant differences between any of these drinking-defined groups and non-drinkers on any of the tasks, not even for the heaviest drinking group. One possible explanation for this finding is that the computerized tasks assessed executive functions in a rather basic way. More complex and strategy-based tasks might be important when studying the effects of alcohol. One indication in this direction was that the same tasks assessed at age 11 were not predictive of the further development of alcohol use, while a large literature would support such a relationship [88, 89], and this relationship was indeed found in the same sample with a self-report measure of effortful control. Hence, while rather basic neuropsychological functions appear to not be affected by alcohol use, including relatively heavy alcohol use, in adolescence, more complex and effortful tasks could still reveal this relationship. However, in the same study, no differences between drinkers and abstainers could be related to alcohol use patterns for a variety of more complex neuropsychological tasks. In contrast, one small study indeed found differences between heavy drinkers and controls, but only in girls, and in one domain: visuospatial functioning [87].

Taken together, although the negative relationship between alcohol use and cognitive functioning in adolescence appears to be well-established, there is little compelling evidence for significant cognitive deficits as a result of adolescent heavy drinking. The effects of alcohol on the developing brain might be more subtle than assumed thus far. This might also be related to the flexibility of the adolescent brain, which also shows stronger recovery after abstinence in heavy episodic drinking adolescents [90] than what is usually found in adult alcohol-dependent patients (review [91]). More research is needed to understand what influence alcohol has on the developing brain and how this could affect functioning in daily life. Possibly, there is a reciprocal effect where weaknesses in effective impulse control might be predictive of later AUD, and heavy drinking prospectively predicting increases in impulsivity [92]. A similar reciprocal relationship has been found for behavioral dishinhibition and early alcohol use onset [93].

### Neurocognitive Studies

Research has shown structural brain differences between heavy substance users and controls [84•]. In addition, emerging data suggest that alterations in activity in specific brain regions (e.g., inferior frontal gyrus) on cognitive control tasks may represent a phenotype of current and future heavy drinking in adolescent participants [94••]. Furthermore, longitudinal studies have shown that atypical brain responses during response inhibition are predictive of later alcohol use [95]. Surprisingly, however, few neurocognitive studies have assessed the effect of alcohol misuse on behavioral control (as assessed using tasks such as the Go/NoGo and Stop-Signal task), or attentional control (as assessed using Stroop, Flanker, or Simon tasks) in adolescence (i.e., ages between 14 and 19).

To the best of our knowledge, only one study examined the effect of prolonged heavy drinking on response inhibition in adolescents. Wetherill and colleagues [96••] conducted a longitudinal study, in which fMRI data were acquired during performance on a Go/NoGo task. Data were collected at baseline from adolescents, aged 14, before the onset of heavy drinking, and then again 3 years later. Results showed that, youth who transitioned into heavy drinking compared with continuous non-drinkers, showed reduced activations at baseline in bilateral prefrontal cortex for the stop vs. go contrast. This response reversed after the onset of heavy drinking [96••]. This is an important finding and is in line with reports in university students showing reduced activations in regions implicated in inhibitory control following acute alcohol administration [97–99], as well as reports showing differential responses in the NoGo P3 ERP as a function of heavy or binge drinking in similar populations [100, 101].

Given the finding that prefrontal cortical gray matter thickness of adolescent females aged 16–19, relative to their male counterparts, is correlated with poorer performance on a color-word interference task [102•], studies should also explore possible gender differences in the impact of alcohol on cognitive control processes. Finally, one interesting prospective study in college students assessed ERPs before and after the onset of binge-drinking behavior [103] and found strong effects of binge drinking in early and global ERP components, suggesting alcohol-related effects on basic and high-level cognitive processes. However, it is unclear to what extent these differences would lead to measurable behavioral effects on neuropsychological tasks and what the long-term effects of (continued) binge drinking would be.

## Conclusion

We reviewed the literature on the effects of adolescent substance use on (neuro)cognitive functions, from a broad dual-process perspective. From this perspective, adolescent substance use would be associated with increasingly strong automatically activated appetitive reactions to substance-related cues, expressed as cue reactivity in the brain, and as attentional bias, approach bias, and memory associations in behavioral tasks. In addition, adolescent substance use is thought to negatively affect the still developing executive or cognitive control functions. Overall, we found many more studies regarding the first set of predictions, which generally supported the idea that adolescent substance users demonstrate substance-related cognitive biases. The impact of these biases on subsequent addictive behavior was found to depend on cognitive control functions, although it should be noted that this was primarily found in high-risk adolescents (sampled from special education), and less so in samples from regular education. Cue-elicited brain activation was also found in adolescent heavy substance users, and in some studies, this predicted subsequent escalation of use. In addition, some studies indicated that the impact of substance-related cues on behavior tended to be stronger after acute alcohol use.

Regarding hypothesized negative effects of alcohol and substance use on executive functions, at a behavioral level there is much less evidence of strong alcohol or substance induced neuropsychological impairments than often thought and the evidence appears to be much weaker here than evidence for the reverse relationship (relatively weak executive functions predicting later problems with substances, as earlier reviews indicated [88, 89]). Neurocognitive studies do find differences between heavy or dependent substance users and controls [84•], but the direction of this effect is often unclear. Also, given the relatively robust reverse relationship, one cannot rule out the possibility that most differences are premorbid to substance involvement. Ongoing large longitudinal studies using neurocognitive measures will tell us more about the effects of alcohol and other substances on the development of cognitive control functions.

Currently, a fair conclusion, based on a review of the literature, appears to be that the effect of alcohol and other substances is primarily found in stronger automatically activated appetitive responses to substance cues, and that these reactions are likely to contribute to the development of problems especially in youth with relatively weak executive control functions (as a premorbid factor). Meta-analysis (including both adolescent and mostly young adult samples) concluded that both implicit and explicit measures (e.g., expectancies and motives) predict unique variance in substance use (mostly alcohol) [104, 105]. Further, this association may be enhanced by acute effects of the substance and perhaps also in the long run by prolonged heavy substance use, and may differ by gender or other subgroupings (e.g., children of alcoholic parents). Imaging and psychophysiological studies are needed that more directly focus on comparing participants of varying ages on their automatic motivational, attentional, and associative responses to addictive stimuli and their ability and tendency to control those responses. Such studies could complement psychological studies by providing inferential support and more detailed constraints for cognitive models, but also by revealing unexpected patterns that may open up new lines of research.

Importantly, environmental factors may also moderate the impact of individual risk factors, such as strong appetitive reactions and weak control: parenting has also been found to moderate the impact of these predictors on subsequent addictive behaviors. And also in the larger picture, automatically activated reactions to substance cues, as assessed with implicit or indirect measures or with neurocognitive indices of cue reactivity, provide only one piece of the puzzle, and in some cases social cues (e.g., peer substance use) are a much stronger predictor than implicit processes (e.g., [106]). Hence, the literature provides evidence that implicit or automatically triggered motivational processes play a role in adolescent substance use, especially among those with relatively weak executive control functions, but other factors such as social (peers and parents), contextual and cultural influences provide other important pieces in the larger puzzle of adolescent substance abuse.

Regarding treatment implications, both implicit and explicit cognitions can be targeted in treatment (see for a review [107••]), but the effects most likely depend on motivation to change, which is an important issue in adolescent substance use problems. Motivation to change could be enhanced, for example with motivational interviewing [108], together with motivation for training or treatment. In addition, positive findings have been reported for targeted personality-based interventions in adolescents [109••]. In conclusion, there is some support for the general perspective from dual process models (at a descriptive level), that in adolescent substance use, the combination of automatically triggered or implicit reactions to substance cues and relatively weak cognitive control processes play a role, with the first factor developing after substance use and the second factor more as a premorbid factor. Intervention methods aimed at adolescent substance users may benefit from this developing knowledge, while at the same time it is important to acknowledge that from a broader perspective other processes such as peer influence are important too.

## References

[1.•] Bechara A (2005). Decision making, impulse control and loss of willpower to resist drugs: a neurocognitive perspective. Nat Neurosci.

[2] Stacy AW, Wiers RW (2010). Implicit cognition and addiction: a tool for explaining paradoxical behavior. Annu Rev Clin Psychol.

[3.•] Wiers RW, Bartholow BD, van den Wildenberg E, Thush C, Engels RC, Sher KJ (2007). Automatic and controlled processes and the development of addictive behaviors in adolescents: a review and a model. Pharmacol Biochem Behav.

[4.••] Munakata Y, Herd SA, Chatham CH, Depue BE, Banich MT, O’Reilly RC (2011). A unified framework for inhibitory control. Trends Cogn Sci (Regul Ed).

[5] Steinberg L (2005). Cognitive and affective development in adolescence. Trends Cogn Sci (Regul Ed).

[6] Giedd JN, Blumenthal J, Jeffries NO, Castellanos FX, Liu H, Zijdenbos A (1999). Brain development during childhood and adolescence: a longitudinal MRI study. Nat Neurosci.

[7] Lenroot RK, Giedd JN (2010). Sex differences in the adolescent brain. Brain Cogn.

[8] Gogtay N, Giedd JN, Lusk L, Hayashi KM, Greenstein D, Vaituzis AC (2004). Dynamic mapping of human cortical development during childhood through early adulthood. Proc Natl Acad Sci U S A.

[9.•] Quevedo KM, Benning SD, Gunnar MR, Dahl RE (2009). The onset of puberty: effects on the psychophysiology of defensive and appetitive motivation. Dev Psychopathol.

[10] Hardee JE, Weiland BJ, Nichols TE, Welsh RC, Soules ME, Steinberg DB, et al. Development of impulse control circuitry in children of alcoholics. Biol Psychiatry 2014. 10.1016/j.biopsych.2014.03.005 PMC4163541 24742620

[11.••] Crone EA, Dahl RE (2012). Understanding adolescence as a period of social–affective engagement and goal flexibility. Nat Rev Neurosci.

[12] Bava S, Tapert SF (2010). Adolescent brain development and the risk for alcohol and other drug problems. Neuropsychol Rev.

[13] Casey B, Jones RM (2010). Neurobiology of the adolescent brain and behavior: implications for substance use disorders. J Am Acad Child Adolescent Psychiatry.

[14.••] Gladwin TE, Figner B, Crone EA, Wiers RW (2011). Addiction, adolescence, and the integration of control and motivation. Dev Cognitive Neurosci.

[15] Field M, Wiers RW, Christiansen P, Fillmore MT, Verster JC (2010). Acute alcohol effects on inhibitory control and implicit cognition: implications for loss of control over drinking. Alcohol Clin Exp Res.

[16] Keren G, Schul Y (2009). Two is not always better than one a critical evaluation of two-system theories. Perspect Psychol Sci.

[17.•] Cunningham WA, Zelazo PD (2007). Attitudes and evaluations: a social cognitive neuroscience perspective. Trends Cogn Sci (Regul Ed).

[18.••] Hazy TE, Frank MJ, O’Reilly RC (2006). Banishing the homunculus: making working memory work. Neuroscience.

[19] Noël X, Brevers D, Bechara A (2013). A neurocognitive approach to understanding the neurobiology of addiction. Curr Opin Neurobiol.

[20.••] Koob GF, Volkow ND (2009). Neurocircuitry of addiction. Neuropsychopharmacology.

[21] Chassin L, Sher KJ, Hussong A, Curran P (2013). The developmental psychopathology of alcohol use and alcohol disorders: research achievements and future directions. Dev Psychopathol.

[22] van Holst RJ, Lemmens JS, Valkenburg PM, Peter J, Veltman DJ, Goudriaan AE (2012). Attentional bias and disinhibition toward gaming cues are related to problem gaming in male adolescents. J Adolesc Health.

[23] Hommer RE, Seo D, Lacadie CM, Chaplin TM, Mayes LC, Sinha R (2013). Neural correlates of stress and favorite‐food cue exposure in adolescents: a functional magnetic resonance imaging study. Hum Brain Mapp.

[24] Fernie G, Peeters M, Gullo MJ, Christiansen P, Cole JC, Sumnall H (2013). Multiple behavioural impulsivity tasks predict prospective alcohol involvement in adolescents. Addiction.

[25] Pieters S, Burk WJ, Van der Vorst H, Engels RC, Wiers RW. Impulsive and reflective processes related to alcohol use in young adolescents. Frontiers Psychiatry 2014;5. 10.3389/fpsyt.2014.00056 PMC4033066 24904439

[26] Pieters S, Van Der Vorst H, Burk WJ, Schoenmakers TM, Van Den Wildenberg E, Smeets HJ (2011). The effect of the OPRM1 and DRD4 polymorphisms on the relation between attentional bias and alcohol use in adolescence and young adulthood. Dev Cognitive Neurosci.

[27] Cousijn J, Watson P, Koenders L, Vingerhoets W, Goudriaan A, Wiers R (2013). Cannabis dependence, cognitive control and attentional bias for cannabis words. Addict Behav.

[28.•] van Hemel-Ruiter ME, de Jong PJ, Ostafin BD, Wiers RW (2015). Reward sensitivity, attentional bias, and executive control in early adolescent alcohol use. Addict Behav.

[29] van Hemel-Ruiter ME, de Jong PJ, Oldehinkel AJ, Ostafin BD (2013). Reward-related attentional biases and adolescent substance use: the TRAILS study. Psychol Addict Behav.

[30] Larsen H, Kong G, Becker D, Cousijn J, Boendermaker W, Cavallo D, et al. Implicit motivational processes underlying smoking in American and Dutch adolescents. Frontiers Psychiatry 2014;5. 10.3389/fpsyt.2014.00051 PMC4032927 24904435

[31] Janssen T, Larsen H, Vollebergh WA, Wiers RW. Longitudinal Relations between Cognitive Bias and Adolescent Alcohol Use. Addict Behav 2014;In press. 10.1016/j.addbeh.2014.11.018 25480559

[32] Willem L, Vasey MW, Beckers T, Claes L, Bijttebier P (2013). Cognitive biases and alcohol use in adolescence and young adulthood: the moderating role of gender, attentional control and inhibitory control. Personal Individ Differ.

[33] Field M, Christiansen P, Cole J, Goudie A (2007). Delay discounting and the alcohol Stroop in heavy drinking adolescents. Addiction.

[34] Cox WM, Fadardi JS, Pothos EM (2006). The addiction-Stroop test: theoretical considerations and procedural recommendations. Psychol Bull.

[35.•] MacLeod C, Mathews A, Tata P (1986). Attentional bias in emotional disorders. J Abnorm Psychol.

[36] Ataya AF, Adams S, Mullings E, Cooper RM, Attwood AS, Munafò MR (2012). Internal reliability of measures of substance-related cognitive bias. Drug Alcohol Depend.

[37.•] Zvielli A, Bernstein A, Koster EH. Temporal dynamics of attentional bias. Clin Psychol Sci 2014:2167702614551572. **Potentially important and promising new scoring method for notoriously unreliable probe tests (see reference #36)**.

[38] Price RB, Kuckertz JM, Siegle GJ, Ladouceur CD, Silk JS, Ryan ND, et al. Empirical recommendations for improving the stability of the dot-probe task in clinical research. Psychol Assessment 2014;In press. 10.1037/pas0000036 PMC4442069 25419646

[39] Zetteler JI, Stollery BT, Weinstein AM, Lingford-Hughes AR (2006). Attentional bias for alcohol-related information in adolescents with alcohol-dependent parents. Alcohol Alcohol.

[40] Wiers RW, Gunning WB, Sergeant JA (1998). Do young children of alcoholics hold more positive or negative alcohol‐related expectancies than controls?. Alcohol Clin Exp Res.

[41] Peeters M, Wiers RW, Monshouwer K, Schoot R, Janssen T, Vollebergh WA (2012). Automatic processes in at‐risk adolescents: the role of alcohol‐approach tendencies and response inhibition in drinking behavior. Addiction.

[42.•] Peeters M, Monshouwer K, Schoot RA, Janssen T, Vollebergh WA, Wiers RW (2013). Automatic processes and the drinking behavior in early adolescence: a prospective study. Alcohol Clin Exp Res.

[43] Pieters S, Burk WJ, Van der Vorst H, Wiers RW, Engels RC (2012). The moderating role of working memory capacity and alcohol‐specific rule‐setting on the relation between approach tendencies and alcohol use in young adolescents. Alcohol Clin Exp Res.

[44] van Hemel-Ruiter ME, de Jong PJ, Wiers RW (2011). Appetitive and regulatory processes in young adolescent drinkers. Addict Behav.

[45.•] Cousijn J, Goudriaan AE, Wiers RW (2011). Reaching out towards cannabis: approach‐bias in heavy cannabis users predicts changes in cannabis use. Addiction.

[46.•] Wiers R, Rinck M, Dictus M, Van Den Wildenberg E (2009). Relatively strong automatic appetitive action‐tendencies in male carriers of the OPRM1 G‐allele. Genes Brain Behav.

[47.•] De Houwer J, Crombez G, Baeyens F, Hermans D (2001). On the generality of the affective Simon effect. Cogn Emo.

[48.•] Rinck M, Becker ES (2007). Approach and avoidance in fear of spiders. J Behav Ther Exp Psychiatry.

[49.•] De Houwer J, Musch J, Klauer K, Musch J, Klauer K (2003). A structural analysis of indirect measures of attitudes. The psychology of evaluation. Affective processes in cognition and emotion.

[50] Field M, Caren R, Fernie G, De Houwer J (2011). Alcohol approach tendencies in heavy drinkers: comparison of effects in a relevant stimulus–response compatibility task and an approach/avoidance Simon task. Psychol Addict Behav.

[51] Wiers RW, Gladwin TE, Rinck M. Should we train alcohol-dependent patients to avoid alcohol? Commentary on Spruyt et al. “On the predictive validity of automatically activated approach/avoidance tendencies in abstaining alcohol-dependent patients”. Frontiers Psychiatry 2013;4(33). 10.3389/fpsyt.2013.00033 PMC3660696 23734131

[52.•] Ames SL, Sussman S, Dent CW, Stacy AW (2005). Implicit cognition and dissociative experiences as predictors of adolescent substance use. Am J Drug Alcohol Abuse.

[53] Ames SL, Grenard JL, Thush C, Sussman S, Wiers RW, Stacy AW (2007). Comparison of indirect assessments of association as predictors of marijuana use among at-risk adolescents. Exp Clin Psychopharmacol.

[54] Belles S, Budde A, Moesgen D, Klein M (2011). Parental problem drinking predicts implicit alcohol expectancy in adolescents and young adults. Addict Behav.

[55] O'Connor RM, Lopez-Vergara HI, Colder CR (2012). Implicit cognition and substance use: the role of controlled and automatic processes in children. J Stud Alcohol Drugs.

[56.•] Grenard JL, Ames SL, Wiers RW, Thush C, Sussman S, Stacy AW (2008). Working memory capacity moderates the predictive effects of drug-related associations on substance use. Psychol Addict Behav.

[57] Thush C, Wiers RW, Ames SL, Grenard JL, Sussman S, Stacy AW (2007). Apples and oranges? Comparing indirect measures of alcohol-related cognition predicting alcohol use in at-risk adolescents. Psychol Addict Behav.

[58] Thush C, Wiers RW (2007). Explicit and implicit alcohol-related cognitions and the prediction of future drinking in adolescents. Addict Behav.

[59] Thush C, Wiers RW, Ames SL, Grenard JL, Sussman S, Stacy AW (2008). Interactions between implicit and explicit cognition and working memory capacity in the prediction of alcohol use in at-risk adolescents. Drug Alcohol Depend.

[60] Van Der Vorst H, Krank M, Engels RC, Pieters S, Burk WJ, Mares SH (2013). The mediating role of alcohol‐related memory associations on the relation between perceived parental drinking and the onset of adolescents’ alcohol use. Addiction.

[61.•] Sherman SJ, Chassin L, Presson C, Seo D, Macy JT, Sherman SJ (2009). The intergenerational transmission of implicit and explicit attitudes toward smoking: predicting adolescent smoking initiation. J Exp Soc Psychol.

[62] O'Connor RM, Fite PJ, Nowlin PR, Colder CR (2007). Children's beliefs about substance use: an examination of age differences in implicit and explicit cognitive precursors of substance use initiation. Psychol Addict Behav.

[63] Pieters S, van der Vorst H, Engels RC, Wiers RW (2010). Implicit and explicit cognitions related to alcohol use in children. Addict Behav.

[64] Coronges K, Stacy AW, Valente TW (2011). Social network influences of alcohol and marijuana cognitive associations. Addict Behav.

[65] Thush C, Wiers RW, Moerbeek M, Ames SL, Grenard JL, Sussman S (2009). Influence of motivational interviewing on explicit and implicit alcohol-related cognition and alcohol use in at-risk adolescents. Psychol Addict Behav.

[66.•] Greenwald AG, McGhee DE, Schwartz JL (1998). Measuring individual differences in implicit cognition: the implicit association test. J Pers Soc Psychol.

[67.•] Conrey FR, Sherman JW, Gawronski B, Hugenberg K, Groom CJ (2005). Separating multiple processes in implicit social cognition: the quad model of implicit task performance. J Pers Soc Psychol.

[68.•] Tapert SF, Cheung EH, Brown GG, Frank LR, Paulus MP, Schweinsburg AD (2003). Neural response to alcohol stimuli in adolescents with alcohol use disorder. Arch Gen Psychiatry.

[69] Dager AD, Anderson BM, Rosen R, Khadka S, Sawyer B, Jiantonio‐Kelly RE (2014). Functional magnetic resonance imaging (fMRI) response to alcohol pictures predicts subsequent transition to heavy drinking in college students. Addiction.

[70] Rubinstein ML, Luks TL, Dryden WY, Rait MA, Simpson GV (2011). Adolescent smokers show decreased brain responses to pleasurable food images compared with nonsmokers. Nicotine Tob Res.

[71] Bunge SA (2004). How we use rules to select actions: a review of evidence from cognitive neuroscience. Cognitive Affective Behav Neurosci.

[72] Korucuoglu O, Gladwin TE, Wiers RW (2014). Preparing to approach or avoid alcohol: EEG correlates, and acute alcohol effects. Neurosci Lett.

[73.•] Ames SL, Wong SW, Bechara A, Cappelli C, Dust M, Grenard JL (2014). Neural correlates of a Go/NoGo task with alcohol stimuli in light and heavy young drinkers. Behav Brain Res.

[74] Ames SL, Grenard JL, Stacy AW, Xiao L, He Q, Wong SW (2013). Functional imaging of implicit marijuana associations during performance on an implicit association test (IAT). Behav Brain Res.

[75] Cousijn J, Goudriaan AE, Ridderinkhof KR, van den Brink W, Veltman DJ, Wiers RW (2012). Approach-bias predicts development of cannabis problem severity in heavy cannabis users: results from a prospective FMRI study. PLoS One.

[76] Ames SL, Grenard JL, He Q, Stacy AW, Wong SW, Xiao L (2014). Functional imaging of an alcohol‐implicit association test (IAT). Addict Biol.

[77.•] Gladwin TE, ter Mors-Schulte MH, Ridderinkhof KR, Wiers RW. Medial parietal cortex activation related to attention control involving alcohol cues. Frontiers Psychiatry 2013;4. **In an alcohol cued attention task, brain activation in the precuneus/posterior cingulate was found to increase when attention had to be directed towards a distracting alcoholic cue, and risky drinking was associated with weaker activation near this region of activation. This may indicate that the role of the precuneus is to attempt to control the influence of alcoholic cues on cognition**. 10.3389/fpsyt.2013.00174 PMC3868991 24391604

[78.•] Korucuoglu O, Gladwin TE, Wiers RW (2015). Alcohol-induced changes in conflict monitoring and error detection as predictors of alcohol use in late adolescence. Neuropsychopharmacology.

[79] Schuckit MA (1994). Low level of response to alcohol as a predictor of future alcoholism. Am J Psychiatry.

[80] Frijda NH. The emotions: Cambridge University Press; 1986.

[81] O'Doherty JP, Hampton A, Kim H (2007). Model‐based fMRI and its application to reward learning and decision making. Ann N Y Acad Sci.

[82] Vollstädt‐Klein S, Loeber S, Richter A, Kirsch M, Bach P, von der Goltz C (2012). Validating incentive salience with functional magnetic resonance imaging: association between mesolimbic cue reactivity and attentional bias in alcohol‐dependent patients. Addict Biol.

[83] Gladwin TE, Wiers RW (2012). Alcohol‐related effects on automaticity due to experimentally manipulated conditioning. Alcohol Clin Exp Res.

[84.•] Jacobus J, Tapert SF (2013). Neurotoxic effects of alcohol in adolescence. Annu Rev Clin Psychol.

[85.•] Lisdahl KM, Gilbart ER, Wright NE, Shollenbarger S. Dare to delay? The impacts of adolescent alcohol and marijuana use onset on cognition, brain structure, and function. Frontiers Psychiatry 2013;4. Review on how substance use in adolescence is related to cognition and brain anatomy and function. 10.3389/fpsyt.2013.00053 PMC3696957 23847550

[86] Boelema SR. Adolescent alcohol use. A longitudinal study of its effect on cognitive functioning. Utrecht University: Unpublished doctoral thesis; 2014.

[87] Squeglia LM, Spadoni AD, Infante MA, Myers MG, Tapert SF (2009). Initiating moderate to heavy alcohol use predicts changes in neuropsychological functioning for adolescent girls and boys. Psychol Addict Behav.

[88] Verdejo-García A, Pérez-García M (2007). Ecological assessment of executive functions in substance dependent individuals. Drug Alcohol Depend.

[89] De Wit H (2009). Impulsivity as a determinant and consequence of drug use: a review of underlying processes. Addict Biol.

[90] Winward JL, Hanson KL, Bekman NM, Tapert SF, Brown SA (2014). Adolescent heavy episodic drinking: neurocognitive functioning during early abstinence. J Int Neuropsychol Soc.

[91] Schulte MH, Cousijn J, den Uyl TE, Goudriaan AE, van den Brink W, Veltman DJ (2014). Recovery of neurocognitive functions following sustained abstinence after substance dependence and implications for treatment. Clin Psychol Rev.

[92] White HR, Marmorstein NR, Crews FT, Bates ME, Mun E, Loeber R (2011). Associations between heavy drinking and changes in impulsive behavior among adolescent boys. Alcohol Clin Exp Res.

[93] Hicks BM, Durbin CE, Blonigen DM, Iacono WG, McGue M (2012). Relationship between personality change and the onset and course of alcohol dependence in young adulthood. Addiction.

[94.••] Whelan R, Watts R, Orr CA, Althoff RR, Artiges E, Banaschewski T (2014). Neuropsychosocial profiles of current and future adolescent alcohol misusers. Nature.

[95] Norman AL, Pulido C, Squeglia LM, Spadoni AD, Paulus MP, Tapert SF (2011). Neural activation during inhibition predicts initiation of substance use in adolescence. Drug Alcohol Depend.

[96.••] Wetherill RR, Squeglia LM, Yang TT, Tapert SF (2013). A longitudinal examination of adolescent response inhibition: neural differences before and after the initiation of heavy drinking. Psychopharmacol (Berl).

[97] Schuckit MA, Tapert S, Matthews SC, Paulus MP, Tolentino NJ, Smith TL (2012). fMRI differences between subjects with low and high responses to alcohol during a stop signal task. Alcohol Clin Exp Res.

[98] Anderson BM, Stevens MC, Meda SA, Jordan K, Calhoun VD, Pearlson GD (2011). Functional imaging of cognitive control during acute alcohol intoxication. Alcohol Clin Exp Res.

[99] Nikolaou K, Critchley H, Duka T (2013). Alcohol affects neuronal substrates of response inhibition but not of perceptual processing of stimuli signalling a stop response. PLoS One.

[100] Petit G, Kornreich C, Noël X, Verbanck P, Campanella S (2012). Alcohol-related context modulates performance of social drinkers in a visual Go/No-Go task: a preliminary assessment of event-related potentials. PLoS One.

[101] López‐Caneda E, Cadaveira F, Crego A, Gómez‐Suárez A, Corral M, Parada M (2012). Hyperactivation of right inferior frontal cortex in young binge drinkers during response inhibition: a follow‐up study. Addiction.

[102.•] Squeglia LM, Sorg SF, Schweinsburg AD, Wetherill RR, Pulido C, Tapert SF (2012). Binge drinking differentially affects adolescent male and female brain morphometry. Psychopharmacol (Berl).

[103] Maurage P, Pesenti M, Philippot P, Joassin F, Campanella S (2009). Latent deleterious effects of binge drinking over a short period of time revealed only by electrophysiological measures. J Psychiatry Neurosci.

[104] Reich RR, Below MC, Goldman MS (2010). Explicit and implicit measures of expectancy and related alcohol cognitions: a meta-analytic comparison. Psychol Addict Behav.

[105] Rooke SE, Hine DW, Thorsteinsson EB (2008). Implicit cognition and substance use: a meta-analysis. Addict Behav.

[106] Larsen H, Engels RC, Wiers RW, Granic I, Spijkerman R (2012). Implicit and explicit alcohol cognitions and observed alcohol consumption: three studies in (semi) naturalistic drinking settings. Addiction.

[107.••] Wiers RW, Gladwin TE, Hofmann W, Salemink E, Ridderinkhof KR (2013). Cognitive bias modification and cognitive control training in addiction and related psychopathology mechanisms, clinical perspectives, and ways forward. Clin Psychol Sci.

[108] Jensen CD, Cushing CC, Aylward BS, Craig JT, Sorell DM, Steele RG (2011). Effectiveness of motivational interviewing interventions for adolescent substance use behavior change: a meta-analytic review. J Consult Clin Psychol.

[109.••] Conrod PJ, Castellanos-Ryan N, Strang J (2010). Brief, personality-targeted coping skills interventions and survival as a non–drug user over a 2-year period during adolescence. Arch Gen Psychiatry.

